# Frequency of hlyA, hlyB, hlyC and hlyD genes in uropathogenic Escherichia coli isolated from UTI patients in Shiraz

**DOI:** 10.3205/dgkh000396

**Published:** 2021-08-30

**Authors:** Heliyaneh Moeinizadeh, Marjan Shaheli

**Affiliations:** 1 Department of Biology, Arsanjan Branch, Islamic Azad University, Arsanjan, Iran

**Keywords:** antibiotic resistance, hly gene, uropathogenic Escherichia coli, urinary tract infection, 16S rRNA

## Abstract

**Background and objectives**: One of the most important causes of urinary tract infections (UTI) is *Escherichia coli*. The infection is mainly due to the uropathogenic strain (UPEC), which has key virulence factors, including hemolysis. In this study, we evaluated the frequency of *hlyA*, *hlyB*, *hlyC* and *hlyD* genes in UPEC strains isolated from clinical samples from Shiraz city, Iran.

**Materials and methods:** 130 urine samples with suspected UTI were collected from Shiraz medical centers and cultured on blood agar and EMB media. Colonies were then characterized by biochemical methods. The genomes were extracted and the presence of hemolysis genes was detected by polymerase chain reaction (PCR) using *hly* gene specific primers and 16S *rRNA*. Drug resistance was assessed by using 10 antibiotic disks in the disk diffusion method, according to CLSI criteria.

**Results:** Out of the 130 collected UTI samples, 100 were identified as UPECs. Within isolates, the *hlyD* gene had the highest frequency – 95% – and *hlyC* had the lowest, with 23%. The frequencies of *hlyA* and *hlyB* genes were calculated as 50% and 43%, respectively. The rates of antibiotic resistance to Azithromycin, Ampicillin, Cefotaxime, Nalidixic Acid, Tetracycline, Trimethoprim-Sulfamethoxazole, Cefepime, Aztreonam, Gentamicin, and Nitrofurantoin were 95%, 86%, 68%, 66%, 65%, 64%, 51%, 46%, 44%, 14%, respectively. 98% of these isolates belonged to the MDR group.

**Conclusion:** This study shows diversity of hemolysis virulence factor in UPECs and unique UPEC drug resistance that would indicate a high antibiotic use in the general population.

## Zusammenfassung

**Hintergrund und Zielsetzung**: Eine der wichtigsten Ursachen für Harnweginfektionen (HWI) ist *Escherichia coli*. Die Infektion ist hauptsächlich auf den uropathogenen Stamm (UPEC) zurückzuführen, der wichtige Virulenzfaktoren einschließlich Hämolyse aufweist. In dieser Studie evaluieren wir die Häufigkeit der *hlyA*, *hlyB*, *hlyC* und *hlyD* Gene in UPEC Stämmen, die aus klinischen Proben in Shiraz isoliert wurden.

**Material und Methode**:130 HWI-verdächtige Urinproben wurden auf Blut Agar und EMB Medien kultiviert. Die Kolonien wurden dann mit biochemischen Methoden charakterisiert. Die Genome wurden extrahiert und das Vorhandensein von Hämolyse Genen wurde durch Polymerasekettenreaktion (PCR), *hly* Gene spezifischer Primer und 16S *rRNA* nachgewiesen. Die Arzneimittelresistenz wurde anhand von 10 Antibiotikaplättchen im Diffusionsverfahren gemäß den CLSI Kriterien bewertet.

**Ergebnisse:** Von den 130 HWI Proben wurden 100 als UPECs identifiziert. Innerhalb der Isolate hatte das *hlyD* Gen die höchste Häufigkeit von 95% und das *hlyC* die niedrigste mit 23%. Die Häufigkeit der *hlyA* und *hlyB* Genen wurde als 50% bzw. 43% berechnet. Die Raten der Antibiotikaresistenz gegen Azithromycin, Ampicillin, Cefotaxim, Nalidixinsäure, Tetracycline, Trimethoprim-Sulfamethoxazole, Cefepime, Aztreonam, Gentamicin und Nitrofurantoin betrugen 95%, 86%, 68%, 66%, 65%, 64%, 51%, 46%, 44% bzw. 14%. 98% dieser Isolation gehörten der MDR-Gruppe an.

**Schlussfolgerungen:** Die Studie zeigt die Vielfalt des Hämolysevirulenzfaktors in UPECs und die UPEC-Arzneimittelresistenz, die auf die häufige Antibiotikaanweneung in der Bevölkerung hinweist.

## Introduction

Urinary tract infection (UTI) is one of the most prevalent bacterial infectious diseases, and is an important factor in the mortality of infants and the elderly [1]. Generally, UTIs are more common among females than males, mainly because of the proximity of the urinary tract and the anus in women. While the male urinary tract is longer than that of in the female and far from the anus, the prostatic fluids in males are also antimicrobially active [2]. Infections of the urinary tract are consistent with a variety of clinical symptoms, affecting the upper urinary tract (kidneys and ureter) and the lower urinary tract (bladder and urethra). It can also appear as symptomatic or non-symptomatic cystitis, pyelonephritis, and in severe cases urosepsis [3]. *Escherichia coli*, from the *Enterobacteriaceae* family, is one of the most common pathogens of the digestive system in human and domestic animals. As a commensal bacterium, it shows a strong association with its host, and it is well known that it causes diseases harshlyas a pote pathoge [4].

Among different strains of *E. coli*, only a few can cause urinary tract infections: these that are known as uropathogenic *Escherichia coli* (UPEC) [5]. To effectively reside in the host's urinary tract, uropathogenic *E. coli* strains express a set of virulence factors, including flagella to move the bacteria, adhesions in order to attach to the tissues and resist the flow of urine [6]. Also, a variety of toxins enable the bacteria to alter the host’s immune responses and escape them [7]. 

One of those toxins is the pore-forming toxin hemolysin, which belongs to the RTX family. As a result, there is a strong connection between increasing urinary tract infection and *hlyA* gene expression in UPEC strains [8].

UTIs have a huge impact on the economy and public health, and alter the quality of life of those who are affected [9].

Antimicrobial resistance is an important problem in global health care. After the advent of penicillin, which was a major breakthrough in antibacterial treatment, different bacteria have developed strong resistance to antibiotics. Bacteria acquire resistance and can transfer it to other species [10]. Increased use and misuse of antimicrobial agents are among the factors that have increased drug resistance. Furthermore, constant travel between countries plays a significant role in the multidrug-resistance (MDR) of many species [11]. MDR strains are increasing worldwide, due to the spread of genes located on mobile genetic elements such as plasmids, integrons, and transposons. The combination of these genes with chromosomally encoded resistance genes often leads to the development of bacterial resistance to the primary classes of antimicrobials [1], [4].

A few studies exist on virulence genes and antibiotic resistance among the UPEC strains causing UTI in Iran. Based on these, this study evaluated the association of UPEC isolates from Shiraz city, Iran, and determined the prevalence of hemolysis genes as well as their correlation with antibiotic resistance.

## Materials and methods

In this descriptive cross-sectional study, a total of 130 urine samples suspected of UTI were obtained from Taghizadegan, Farhangiyan, and Farzanegan laboratories in Shiraz city over a period of six months from February to July, 2019.

Standard bacteriological and biochemical tests were done to identify and isolate the *E. coli* strains. The urine samples were cultured on eosin methylene blue (EMB) agar and blood agar and incubated at 37°C for 24 hours. The colonies with metallic-green color were selected for further phenotypic identification tests, including TSI, Citrate, SIM, Urease, MR-VP, LD and OD. Finally, the confirmed *E. coli* isolates were suspended in skim-milk media to be preserved for further experiments.

### Antimicrobial susceptibility testing

The Kerby-Bauer disk diffusion method was carried out on Muller Hinton agar medium to determine the antibiotic susceptibility of the *E. coli* isolates to cefotaxime (CTX) (30 µg), nalidixic acid (NA) (30 µg), cefepime (FEP) (30 µg), tetracycline (TET) (30 µg), azithromycin (AZM) (15 µg), nitrofurantoin (FM) (300 µg), gentamicin (GM) (10 µg), aztreonam (AZ) (30 µg), ampicillin (AMP) (10 µg) and trimethoprim-sulfamethoxazole (SXT) (1.25/ 23.75 µg), according to the Clinical Laboratory Standard Institute Criteria [12]. The *E. coli* PTCC 1338 strain was used as quality control.

### DNA extraction and detection of virulence factor

The genomic DNA and the hemolysis virulence genes were extracted from 100 UPEC isolates using a DNA extraction kit (CinnaGen Co., Iran). Extracted DNA was kept in skim milk between –50° C and –70° C until it was required for other tests. DNA was amplified using a thermal cycler (Eppendorf, Germany) in a total volume of 25 µL. The liquid mixture consisted of 4 µL DNA template, 12.5 µL Master Mix (2X), 2.5 µL of specific primers (10 pmol) and 6 µL distilled water. A standard polymerase chain reaction (PCR) was performed according to the manufacturer’s instructions (see Table 1 (Tab. 1)). The PCR products were analyzed by electrophoresis on 2% agarose gel along with the 100-bp DNA ladder as a marker. Gels were stained with ethidium bromide and detection of the amplified DNA was conducted using a UV transilluminator (Unico, China).

### Statistical analysis

The statistical analysis was done using SPSS software version 20 (SPSS Inc., Chicago, IL, USA). The *chi*-squared test was used to assess the correlation between variables. A p-value <0.05 was considered significant. 

## Results

### Frequency of virulence genes 

Approximately 100 (90.80%) *E. coli* were confirmed out of the 130 isolates using differential and biochemical tests and the PCR of specific *16S rRNA*. They were proven by the appearance of a 620 bp band on 2% agarose gel.

**Figure 1:**
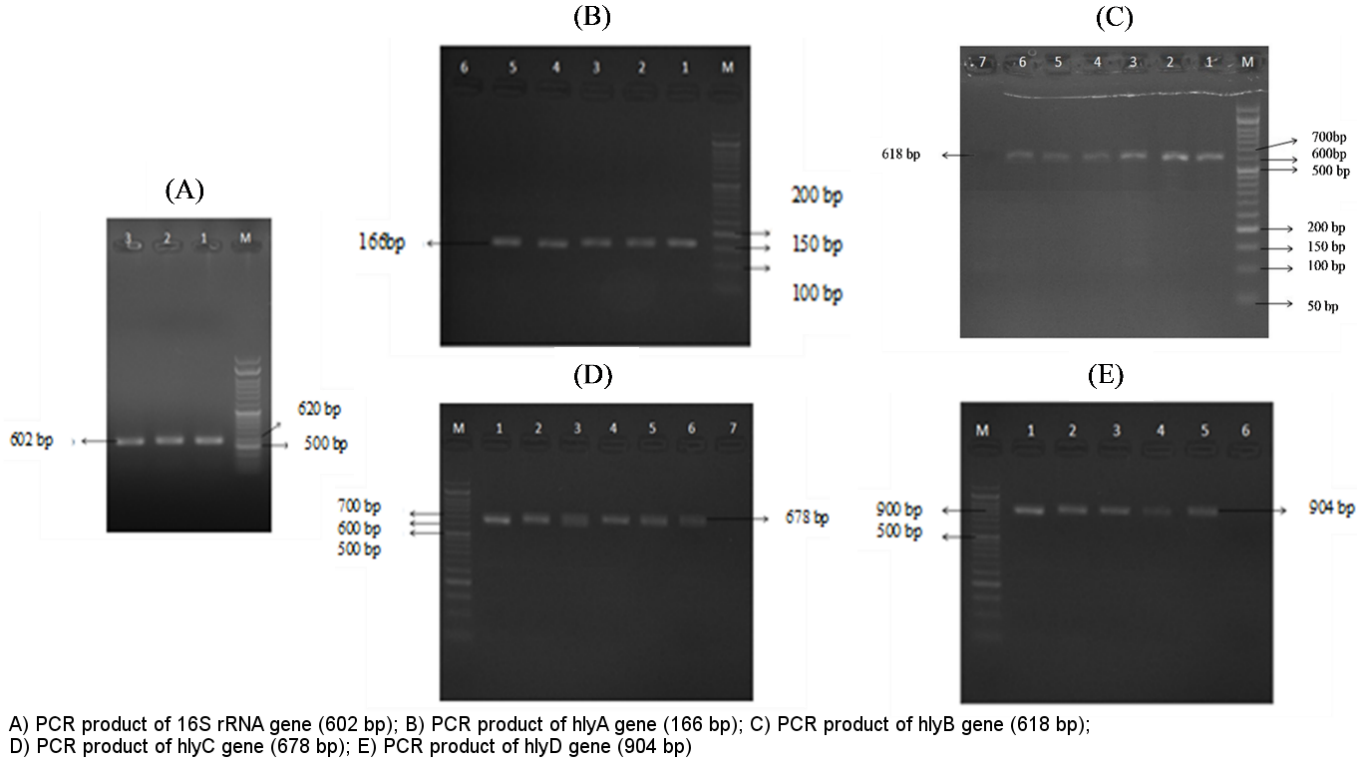
Gel electrophoresis of PCR products

Analysis of hemolysis genes showed that among 100 UPEC isolates, *hlyD* was the most frequent virulence gene, detected in 95% of the isolates, followed by *hlyA* with 50%, *hlyB* with 43%, and *hlyC* with 23% (Figure 1 (Fig. 1)). The PCR products were evaluated on 2% agarose gel.

In the phenotypic study of the *hly* genes, wide genetic diversity was observed and only 19% of the isolates had all the genes of the *hly* operon at the same time, which indicates that the isolates may have undergone a series of mutations.

The present study also evaluated the likelihood of the simultaneous presence of a gene pair. The *chi*-squared test indicates a statistically significant correlation between the simultaneous presence of *hlyC* and *hlyD* genes in study subjects (p<0.05). 

### Antibiotic susceptibility among UPEC isolates 

In this study, the examined isolates showed more than 50% resistance to 70% of the tested antibiotics.

**Figure 2:**
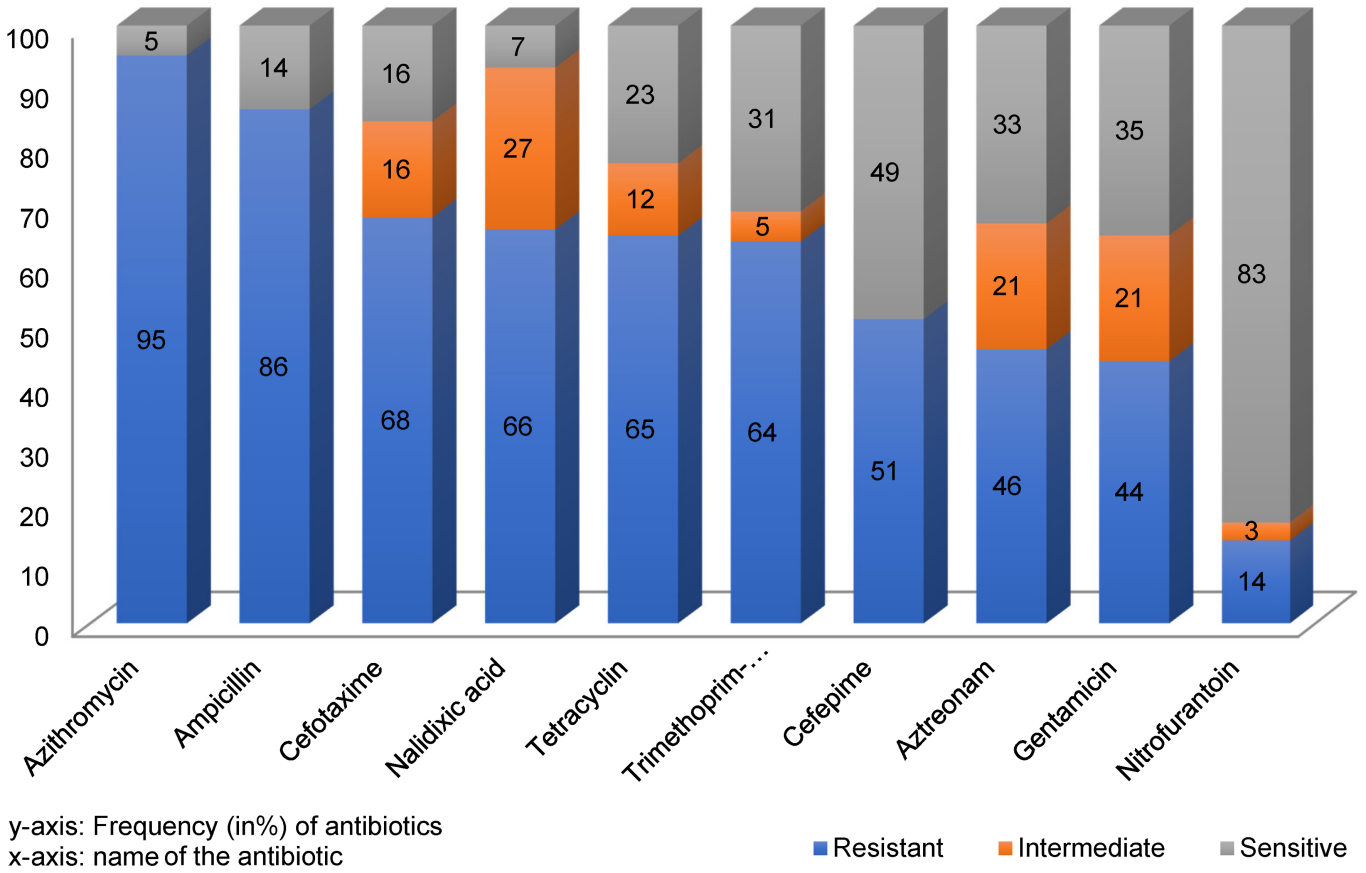
Frequency of antibiotic resistance or susceptibility

The results of the antibiotic susceptibility test showed that Azithromycin (95%) and Ampicillin (86%) had the highest resistance rates. The isolates had the lowest resistance to Gentamicin (44%) and Nitrofurantoin (14%). The prevalence of resistance against Cefotaxime, Nalidixic Acid, Tetracycline, Trimethoprim-Sulfamethoxazole, Cefepime, and Aztreonam was 68%, 66%, 65%, 64%, 51%, and 46%, respectively (Figure 2 (Fig. 2)). 

Based on the one sample T test, the study population became resistant to other antibiotics except Azteronam, gentamicin and nitrofurantine antibiotics and a significant relationship was formed between resistance and microbial population.

 According to the chi-square test, in the study population, there is a significant relationship between resistance and the use of more than one antibiotic. The simultaneous use of these antibiotics or replacement of one for the others had no impact on the treatment result.

This study also shows some unique antibiotic resistance patterns in the studied population. For instance, eight strains demonstrated resistance to all antibiotics. Thus, this finding highlights the importance of knowing the patient’s specific infection, awareness of the diversity of antibiotic resistance and therefore the fact that the medication might not be effective against some infections yet very successful against others.

As shown in Table 2 (Tab. 2), 10 antibiotics from 9 classes of antibiotics were used and 98% of the isolates were verified to be multidrug-resistant (MDR).

In the case of hemolysis genes, although the studied operons were important factors in the invasion of bacteria, our results (Table 3 (Tab. 3)) indicate no significant correlation between the presence or absence of these genes and antibiotic resistance.

## Discussion

In humans, after respiratory tract infections, urinary tract infections are the second most important bacterial infection. In many cases, infection recurrence has been reported, which makes the treatment very difficult. UTI can spread and damage the parenchyma of the kidneys, which leads to kidney failure. Within the UTIs, different clinical steps and complications, such as the stages of diagnosis, management, side effects, and in some cases severity that can cause death, impose a heavy burden on society and health care systems. In addition to these, with increasing resistance to important antimicrobial agents, new pathogenic strains will emerge. These factors can lead to more expensive, resource-consuming ways of controlling these infections. Some studies showed that severe UTIs are due to the existence of a variety of pathogens. Therefore, studies targeting pathogens are of particular importance and might play a crucial role in the production and development of effective treatment or vaccines against such infections. Studying the pathogenic factors involved in UTIs highlights the role of uropathogenic *Escherichia coli* as one of the main bacteria causing UTIs. We may be able to provide solutions to this problem by studying patient-specific antibiotic resistance [2]. 

In the current study, 98% of the UPEC isolates were multidrug-resistant. This finding was consistent with those reported by Spindola et al. [13] and Rehman et al. [14], but differed from reports by Navidinia et al. (26%) [15].

The specific evaluations in Mostafavi’s study showed that resistance to cefotaxime and tetracycline were very high, with 100% and 80%, respectively, followed by 20/24% resistance to gentamycin, 0% to nitrofurantoin, and a 32/60% frequency of *hlyA* gene, so the reducing process of resistance to these antibiotics is similar to the results of this study [16]. 

According to studies by Navidinia [15] and Shabani [17] on UTI patients, the lowest resistances were to gentamicin and nitrofurantoin and the highest were to ampicillin and azithromycin, which is consistent with our study.

Comparison with other study populations indicates statistically different significantly results. For instance, studies performed on Indian [18] or Turkish subjects [19] showed the highest resistance was found to gentamicin and nitrofurantoin, while our study population demonstrated the lowest resistance to these two antibiotics. These comparisons emphasize the importance of geographical status and antibiotic regimens in different medical and health facilities.

In another study, Karami et al. [20] evaluated 205 Swedish one-year-olds and showed that the lowest resistance rates were to gentamicin and nitrofurantoin. Karimi et al. concluded that there was a significant increase in the resistance of uropathogenic *Escherichia coli* to antibiotics, which may be due to the transmission of resistance genes between strains of bacteria over the years. In their study population, they showed a 22% presence of the *hlyA* gene, which is different from our study population.

The simultaneous presence of invasive agents and antibiotic resistance could be involved in the pathogenicity, but the current study did not detect a significant correlation between these two factors in our subjects.

Comparing our results with other reports, a 19% presence of the *hly* gene was compatible with the research of Tarchouna et al. [21] and Staji et al. [22] on Tunisian subjects, but interestingly different from a study population from the northern part of Iran reported by Raespour et al. [23] (60%).

Paniagua-Cotreras et al. [24] concluded that the *hlyA* gene was present in 15.4% of the isolates, and most of these strains were resistant to ampicillin and cefotaxime, the results of which were quite similar to those obtained in this study. In contrast, Moustafa et al. [25] found 11% *hlyA* and 10% *hlyB*, 10% *hlyC*, and 10% *hlyD* genes in 85 stool samples from people with intestinal inflammation at San Diego University in the USA, which contradicts the results of this study. 

In Romania, Cristea et al. [26] reported 47.52% antibiotic resistance to ampicillin and 41.16% to tetracycline. Those authors also found the frequency of *hlyA* and *hlyD* genes to be 12.45% and 44.34%, respectively, which did not agree with the results of our study population. On the other hand, similar to their study, this study showed that *hlyD* has a higher frequency than *hlyA*. Furthermore, in the Cristea et al. study, the MDR value was 35.19%, which is much lower than the MDR in our subjects, i.e., 98%.

Studies done by Ali et al. [27] and Ghosh et al. [28] in 2019 indicated that the frequencies of the *hlyA* gene in Pakistan and the *hly* gene in India were 12% and 10%, respectively, which were different from this study’s results. The resistance rate to cefotaxime, gentamycin, and nitrofurantoin were 100%, 50/57%, and 12/50% respectively, according to Ghosh et al. [28].

In Iraq, Mohammad et al. [29] reported a 100% frequency of the *hlyA* gene, which was much higher than the rate of that gene in the current study. 

In our research, we could not find any similar study on Iranian subjects evaluating the four *hly* genes in UPEC isolates, so we cannot compare our study results with previous ones in similar populations.

## Conclusions

Assessment of UPEC isolates in present study showed that out of 100 *Escherichia coli* isolates from patients suspected of UTI, 19% harbored the four evaluated *hly* genes. 81% of the samples had a hemolysin operon mutation, so that part of the hemolysin structure was lost due to the loss of stated genes. Nevertheless, there is no influence on the etiology of the disease, indicating the existence of other pathogens in UPECs. As the activity of hemolysin could cause destruction of kidney cells and nephropathogenicity, the observed 19% frequency of these four genes in UTI patients should raise the alarm and encourage molecular analysis of these genes in UTI patients to prevent very adverse outcomes, such as kidney failure in UTI patients. 

Evaluation of the results for antibiotic resistance suggests that UTI strains become highly resistant to azithromycin and ampicillin, so patients and physicians should be much more careful in the usage or prescription of these antibiotics in UTIs. In UTIs, alternative antibiotics such as nitrofurantoin and gentamicin might be effective substitutes. It is essential to note that given the high resistance to important antibiotics and the occurrence of 98% multidrug-resistant strains reported here and elsewhere, it is even more important to focus on the existence of resistance genes and *Escherichia coli’s* proclivity for acquiring them through horizontal gene transfer (HGT).

Overall, the noted differences in antibiotic resistance and abundance of hemolysin genes may be due to the influence of routine treatment regimens in each population and the effect of environmental factors and the different pathogenic strains in different regions.

## Notes

### Competing interests

The authors declare that they have no competing interests. This article is an excerpt from a student’s thesis with code 1602922953320951397119700. 

No human samples were used in this article by any of the authors directly.

We received the anonymous samples randomly from the laboratory.

### Funding

Funds were provided by the student Ms. Heliyaneh Moeinizadeh.

### Acknowledgement

We are highly grateful to the Cell Biotechnology Saba Arna knowledge-based company, Shiraz, Iran, for their support in this study. 

This article is part of the MSc thesis by Ms. Heliyaneh Moeinizadeh under the supervision of Dr. M. Shaheli.

## Figures and Tables

**Table 1:**
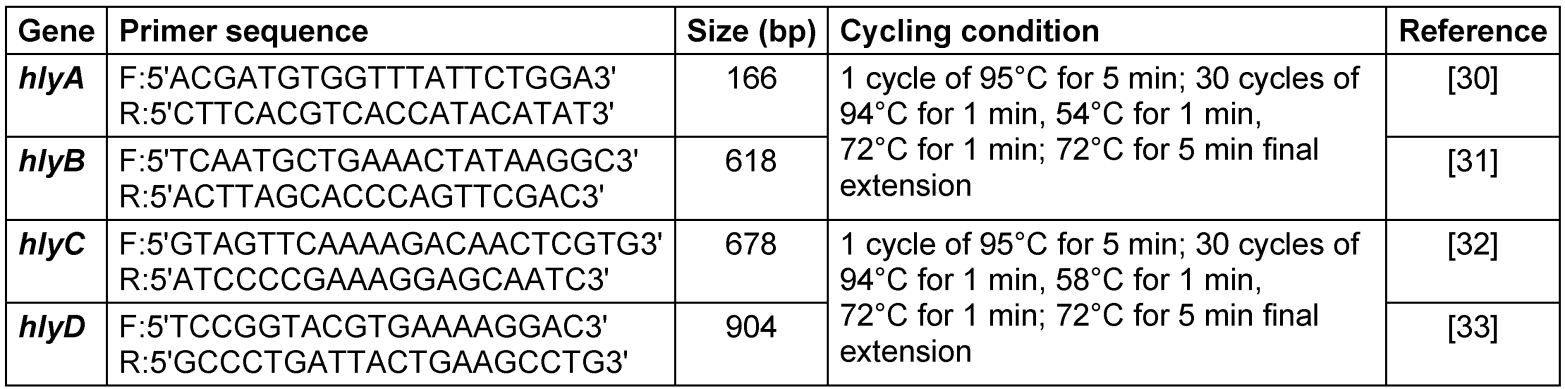
Primers and PCR cycling conditions

**Table 2:**
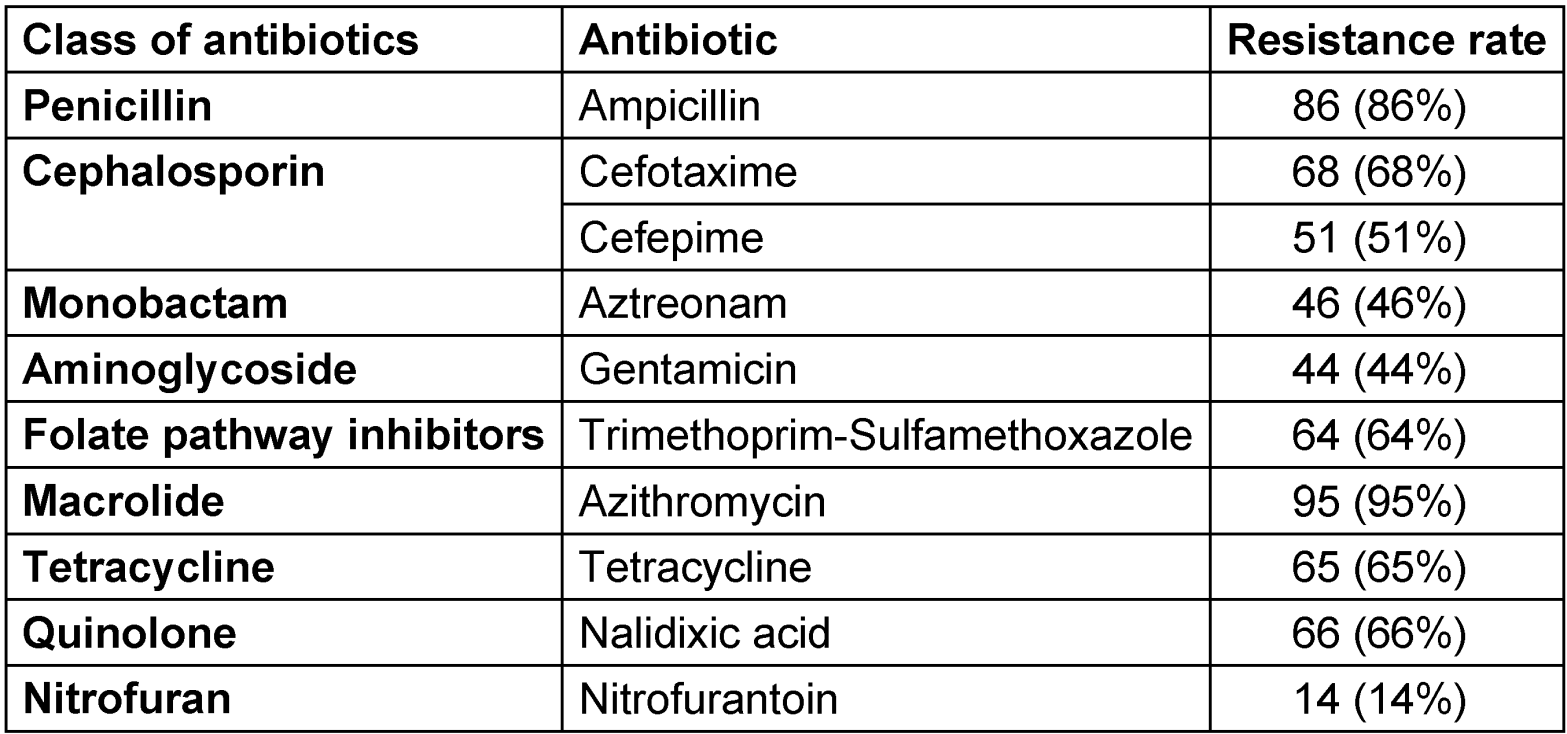
Frequency of antibiotic resistance to different antibiotic classes

**Table 3:**
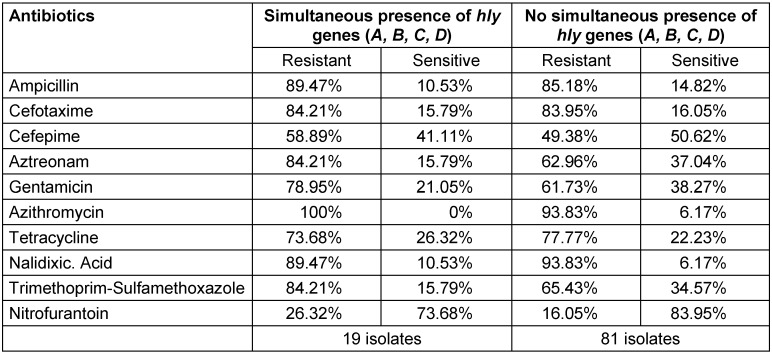
Comparison of the presence or absence of hly genes with antibiotic resistance
